# Liqui-Pellet: the Emerging Next-Generation Oral Dosage Form Which Stems from Liquisolid Concept in Combination with Pelletization Technology

**DOI:** 10.1208/s12249-019-1441-9

**Published:** 2019-06-24

**Authors:** Matthew Lam, Taravat Ghafourian, Ali Nokhodchi

**Affiliations:** 1 Pharmaceutics Research Laboratory, Arundel Building, School of Life Sciences, University of Sussex, Brighton, UK; 2 JMS Building, School of Life Sciences, University of Sussex, Brighton, UK

**Keywords:** liqui-pellet, liquisolid, dissolution enhancement, powder flow, naproxen, solid state analysis

## Abstract

In spite of the major advantages that the liquisolid technology offers, particularly in tackling poor bioavailability of poorly water-soluble drugs (*i.e.*, BCS Class II drugs), there are a few critical drawbacks. The inability of a high liquid load factor, poor flowability, poor compactibility, and an inability to produce a high dose dosage form of a reasonable size for swallowing are major hurdles, hampering this technology from being commercially feasible. An attempt was therefore made to overcome these drawbacks whilst maintaining the liquisolid inherent advantages. This resulted in the emerging next generation of oral dosage forms called the liqui-pellet. All formulations were incorporated into capsules as the final product. Solubility studies of naproxen were conducted in different liquid vehicles, namely polyethylene glycol 200, propylene glycol, Tween 80, Labrafil, Labrasol, and Kolliphor EL. The scanning electron microscopy studies indicated that the liquid vehicle tends to reduce the surface roughness of the pellet. X-ray powder diffraction (XRPD) indicated no significant differences in the crystalline structure or amorphous content between the physical mixture and the liqui-pellet formulation. This was due to the presence of a high concentration of amorphous Avicel in the formulation which overshadowed the crystalline structure of naproxen in the physical mixtures. Flowability and dissolution tests confirmed that this next-generation oral dosage form has excellent flowability, whilst maintaining the typical liquisolid enhanced drug release performance in comparison to its physical mixture counterpart. The liqui-pellet also had a high liquid load factor of 1, where ~ 29% of the total mass was the liquid vehicle. This shows that a high liquid load factor can be achieved in a liqui-pellet without compromising flowability. Overall, the results showed that the poor flowability of a liquisolid formulation could be overcomed with the liqui-pellet, which is believed to be a major advancement into the commercial feasibility of the liquisolid concept.

## INTRODUCTION

Liqui-pellet is an emerging novel oral dosage form, which improves the bioavailability of poorly water-soluble drugs *via* increasing drug release rate in the GIT. The poor drug dissolution rate of water-insoluble drugs is, in fact, a major issue confronting the pharmaceutical industry (1). It is worth pointing out that around 60% of drugs in the market are poorly soluble in gastrointestinal fluids, which is based on biopharmaceutical classification system (BCS), and around 40% of drugs in development are identified as poorly water soluble (2,3).

**Fig. 1:**
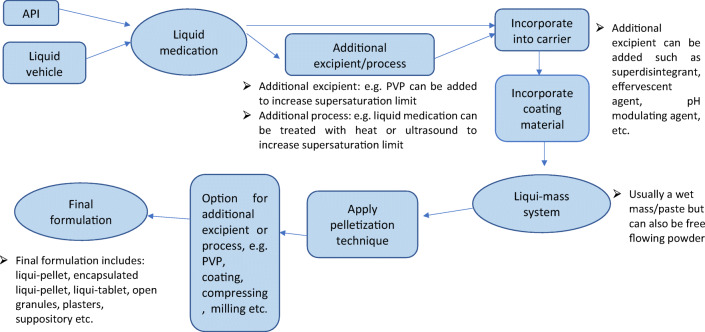
Diagram summarizing the novel liqui-mass system which is used to make liqui-pellet

Liqui-pellet stems from combining liquisolid concept with pelletization technology. It is fundamentally different from liquisolid technology in that it does not fit under the definition of liquisolid system; hence, it is called liqui-pellet instead of liquisolid pellet. Liquisolid formulation is described to be under liquisolid system, which refers to powdered form of liquid medication formulated by transforming liquid lipophilic drugs, or drug suspensions or solutions of water-insoluble drugs in an appropriate non-volatile liquid vehicle into dry looking nonadherent, free-flowing, and readily compressible powder admixtures by incorporating specific carriers and coating materials (4). In this study, the liqui-pellet cannot be described as a liquisolid system because it is not necessarily in powder form and the admixture is not necessarily free-flowing, but rather a cohesive wet mass. The formulation only becomes free-flowing after becoming a pellet. In addition, the purpose of entitling the new dosage form as liqui-pellet is to emphasize the high liquid load factor or high amount of liquid vehicle it is capable of containing. This implies that a high amount of liquid medication can be incorporated into the formulation, which can result in either reduction in dosage weight or increased of API in solubilized state, or both. The new system is described as liqui-mass system, which is summarized in Fig. 1. The liqui-mass system is versatile and different modifications can be applied to it. In this study, the key focus is producing liqui-pellet *via* liqui-mass system. However, it should be noted that this novel technology is still in its infancy; there is a high degree of flexibility for modification regarding liqui-pellet and the liqui-mass composition as shown in Fig. 1.

In order to have a good grasp of liqui-pellet, it is important to understand liquisolid technology. Liquisolid technology’s simplistic approach and cost-effectiveness are desirable when considering manufacturing at a commercial scale (5). In fact, the excipients used are conventional and commonly available in the market (5). In addition to enhanced drug release, the formulation can be manipulated to achieve sustained drug release with a near zero-order release kinetic (6,7). Despite the advantages, it has yet to overcome drawbacks, which hampers it from becoming a commercial product. This is mainly due to major issues such as poor flowability, poor compressibility, and the inability to produce high dose drug without being too bulky and heavy, which is not ideal for swallowing (1,5). The flow property of the liquisolid blend is of critical importance in terms of manufacturing, particularly tablet or capsule form, as flow property determines uniform feed and reproducible filling (8).

In brief, the concept of liquisolid system is comprised of an active pharmaceutical ingredient (API), which is solubilized in a liquid vehicle, forming the liquid medication. This liquid medication is then incorporated into a carrier which is coated with nano-sized coating material to give the admixture of API and excipients a dry, free-flowing, and readily compressible properties (1,9).

Although there are other various technologies confronting the issue of poor drug dissolution rate of water-insoluble drugs, they may require advanced techniques, sophisticated machinery, and complicated technology or may not be cost-effective (5). The other technologies include conversion of crystalline drug into its amorphous state (10), micronization (11–14), solid dispersion (15), co-grinding (16–18), nanosuspension (19,20), self-emulsifying drug delivery system (21,22), and inclusion of drug solution in soft gelatin capsule (23). But in most cases, long-term stability is an issue. For example, on storage, highly amorphous materials can be converted to crystalline state which usually changes drug release profile (24,25).

Since the focus of this study involves combining concept from liquisolid technology with pelletization technology, specifically extrusion-spheronization technology, the understanding of optimal extrudate properties for spheronization and parameters affecting the formation of pellets is prudent. In order to carry out wet extrusion, the material must display sufficient plastic property and cohesiveness to allow shaping and retention of the extrudate. The extrudate for pellet production should be self-lubricating and eventually brittle but not friable (26). Moisture in the powder mass is one of the major factors necessary for providing plasticity for extrusion and spheronization, which have been subjected to much research (27–32). Water content is found to be one of the most important parameters (31). Other factors that can affect pellets’ properties are water/granulating liquid temperature, extrusion speed, spheronization speed, and spheronization duration (30).

The aim of the present study is to make liqui-pellet, which has the inherent advantages from both liquisolid and pelletization technologies. The extrusion-spheronization technique can improve flow property, and the inherent advantages from the liquisolid aspect can enhance the drug release rate. The authors have termed this next-generation oral dosage form as liqui-pellet. This is to distinguish itself from the classical liquisolid compact, to emphasize the high liquid load factor it is capable of, and most importantly, to make clear that it is fundamentally different from liquisolid formulation in that it does not correspond to liquisolid system, but instead to liqui-mass system. The excellent flowability of liqui-pellet means there is more room to increase liquid load factor or the amount of liquid vehicle. The inherent advantages from pelletization technology include reduced risk of side effects due to dose dumping, combining incompatible drugs or drugs with different release profiles in the same dose unit (33), and having good flow property (34).

## MATERIALS AND METHODS

### Materials

Naproxen was obtained from Tokyo Chemical Industry Co (Japan). Other excipients used to prepare the liqui-pellet included microcrystalline cellulose (Avicel PH-101), (FMC Corp., UK); colloidal silicon dioxide (Aerosil 300), (Evonik Industries AG, Hanau, Germany); polyethylene glycol 200 (Fisher Scientific, Leicester, UK); propylene glycol (SAFC, Spain); polysorbate 80 (Tween 80), (Acros, Netherlands); linoleoyl macrogol-6 glycerides (Labrafil), (Gattefosse, Saint Priest, France); caprylocaproyl macrogol-8 glycerides (Labrasol), (Gattefosse, Saint Priest, France); and macrogolglycerol ricinoleate 35 (Kolliphor EL), (BASF SE, Ludwigshafen, Germany). All other reagents and solvent were of analytical grades.

### Solubility Studies

Saturated solubility studies were carried out using 6 different liquid vehicles, *i.e.*, polyethylene glycol 200 (PEG 200), propylene glycol (PG), Tween 80, Labrafil, Labrasol, and Kolliphor EL (the liquid vehicles were selected on the basis of the published articles and their solubilizing effect on the drug (35,36). The primary objective of using various mentioned liquid vehicles was to determine which liquid vehicle was the most suitable liquid vehicle for enhanced released naproxen liqui-pellet formulation. Saturated solutions were prepared by adding excess pure naproxen in a small vial containing 10 ml of specified liquid vehicle. The sample was then left in a bath shaker (OLS Aqua Pro, Grant Instruments Ltd., UK) for 48 h under a constant temperature of 37°C and shaking speed of 40 rpm. The supernatant was then filtered through a pre-heated filter (pore size 0.22 μm, Millex GP, Merck Millipore Ltd., Ireland), and diluted with phosphate buffer solution. This was then analyzed *via* a UV/vis spectrophotometer (Biowave II, Biochrom Ltd., UK) at wavelength 271 nm to determine the concentration of naproxen in each sample. Each test was carried out in triplicates.

### Preparation of Naproxen Liqui-Pellet

The liqui-pellets were prepared by mixing pure naproxen in a chosen liquid vehicle (PEG 200, PG, Tween 80, Labrafil, Labrasol, and Kolliphor EL) using pestle and mortar method. All formulations contained Avicel PH-101 and Aerosil 300 as carrier and coating materials respectively, with a weight ratio of carrier to coating material of 20 (R-value). The liquid load factor for all liqui-pellet formulations was 1. Liquid load factor is the ratio of the weight of liquid medication over the weight of carrier excipient in the formulation admixture. Avicel PH-101 was mixed into the admixture to make sure the wet liquid medication was absorbed by the carrier and not leaving residual in the mortar when transferred into a mixer (Caleva Multitab, Caleva Process Solutions Ltd., UK). The sample was mixed for 10 min at a constant rate of 125 rpm with deionized water added bit by bit to achieve a desirable plasticity for extrusion (Caleva Multitab, Caleva Process Solutions Ltd., UK). The preliminary studies indicated that water content was a crucial factor to achieve extrudate with optimal plasticity for quality spherical pellet after spheronization (Caleva Multitab, Caleva Process Solutions Ltd., UK), which was further supported by the data published in the literature (31). Aerosil 300 was then added into the admixture and further mixed for 10 min before the extrusion-spheronization process. Spheronization was set at an almost constant rotation at 4000 rpm (decrease to 3500 rpm if agglomeration seemed likely or increase to 4500 rpm to increase pellet sphericity); however, in each formulation, spheronization time varied depending on the extrudate plasticity property. Pellets were then placed in an oven under a constant temperature of 50°C overnight to remove water from pellets. Table I shows the details of each formulation with different liquid vehicles. Please note physical mixture pellet was prepared in the same manner as liqui-pellet except liquid vehicle was absent.

**Table I:** Key Formulation Characteristics of the Investigate Liqui-Pellet in Capsule

Formulation	Liquid vehicle	Liquid vehicle concentration (% *w*/*w*)	Mass of carrier (mg)	Mass of coating material (mg)	Liquid load factor	Total weight of 25 mg naproxen liqui-pellet (mg)
Physical mixture pellet			68.97	3.13		90.63
LP-1	PEG 200	29.27	62.50	3.12	1	128.13
LP-2	PG	29.27	62.50	3.12	1	128.13
LP-3	Tween 80	29.27	62.50	3.12	1	128.13
LP-4	Labrafil	29.27	62.50	3.12	1	128.13
LP-5	Labrasol	29.27	62.50	3.12	1	128.13
LP-6	Kolliphor EL	29.27	62.50	3.12	1	128.13

Note, all formulation contains 25 mg of naproxen and the carrier to coating material is at a ratio of 20:1

### Evaluation of Naproxen Liqui-Pellet

#### Assay of Drug Content

Assays were carried out in all naproxen liqui-pellet samples in order to confirm that all formulations contained the expected amount of drug that meets USP requirement of 90–110%. Assays were carried out *via* crushing specified amount of pellets and dissolving the sample in a specified amount of phosphate buffer solution (pH 7.4) for spectrophotometric analysis (Biowave II, Biochrom Ltd., UK) at a wavelength of 271 nm where naproxen can be detected. The same method was used to test pure naproxen powder.

#### Flowability Test on Liqui-Pellet

Techniques of measuring flow property of the liqui-pellet that were used were flow rate in grams per second (Flowability tester, Copley Scientific, UK), angle of repose (Flowability tester, Copley Scientific, UK, and Digimatic height gauge, Mitutoyo, Japan), and Carr’s compressibility index using the SVM tapped density tester (D-63150, Erweka, Germany). The flow rate was measured by recording the weight (g) and time (s) of pellets flowing through a 10-mm diameter orifice. The shutter was applied before funnel became empty of pellets. To determine the angle of repose, the pellets were placed in a funnel with a 10-mm diameter orifice and let the pellets flow onto a 100-mm diameter circular test platform. The digimatic height gauge and micrometer were used to measure the height and diameter of the heap of the sample, so that the angle of repose could be determined. Carr’s compressibility index (CI%) was calculated from the poured (P_b_) and tapped (P_t_) densities using CI equation (Eq. 1). Tapped density was measured using the tapped density tester, which was set for 100 taps. All measurements were done in triplicates.

1 $$ \mathrm{CI}\%=\left({\mathrm{P}}_{\mathrm{t}}\hbox{--} {\mathrm{P}}_{\mathrm{b}}\right)/{\mathrm{P}}_{\mathrm{t}}\times 100 $$

#### Friability Test on Liqui-Pellet

Since there is no official standard for friability test on pellets, friability test was adapted using a similar method used by Hu *et al*. (37). All formulations were tested. Pellets (3 g) and glass beads (3 g) were placed in a Erweka friabilator (D-63150, Erweka, Germany) under constant rotation of 25 rpm for 4 min. Note that the friabilator was sealed in order to prevent pellets leaving the container. Weight of the pellets before and after the friability test was recorded in order to calculate % weight loss.

#### Particle Size Analysis *via* Sieve Method

Sieves (Test sieve, Retsch, Germany) were used to determine the size distributions of all formulations. Pellets (5 g) were sieved under vibration *via* a mechanical shaker (AS 200, Retsch, Germany) for 1 min with an amplitude of 50, then a further 9 min with an amplitude of 40, using 2000, 1000, 850, 500, and 250 μm sieves. The pellets’ yield was determined based on the pellet fraction between 250 and 2000 μm and shown as the % of total pellet weight.

#### Stereoscopic Analysis

Stereoscopic analysis was performed on all formulations using an optical microscope (Nikon Labophot, Nikon, Japan), which was attached to a camera (Panasonic camera WVCL310, Panasonic, Japan). This allowed the mean Feret’s diameter, roundness, and elongation ratio to be calculated using the particle size analysis software V1999 (designed in-house at King’s College London). Note that 100 pellets per formulation were analyzed and the roundness and elongation ratio was calculated using Eqs. 2 and 3 respectively (38).

2 $$ \mathrm{Roundness}={\left(\mathrm{perimeter}\right)}^2/\left(4\times \uppi \times \mathrm{Area}\right) $$

3 $$ \mathrm{Elongation}\ \mathrm{ratio}=\mathrm{Maximum}\ \mathrm{Feret}\ \mathrm{diameter}/\mathrm{Minimum}\ \mathrm{Feret}\ \mathrm{diameter} $$

#### Scanning Electron Microscope Analysis

A scanning electron microscope (Jeol JMS 820, Freising, Germany) was used to observe the morphology of the pellets of each formulation. Each sample was placed in a double-sided carbon tape and sputter-coated with gold using a sputter coater (Edwards S-150 Sputter Coater, Edwards High Vacuum Co. International, USA) before placing in the scanning electron microscope (SEM) machine. The surface structure was then observed and recorded at magnifications of × 80, × 200, and × 800, using the SEM which was operating at 3 kV.

### *In vitro* Drug Release Test

All dissolution tests were carried out using USP paddle method (708-DS Dissolution Apparatus & Cary 60 UV-Vis, Agilent Technologies, USA). The formulations in the form of liqui-pellets in capsules were under constant conditions of 900 ml of dissolution medium, paddle agitation of 50 rpm, and temperature of 37.3 ± 0.5°C. Dissolution medium was either HCl buffer solution of pH 1.2 or phosphate buffer solution of pH 7.4 to simulate gastric fluid and intestinal fluid respectively without enzymes. Absorbance (at 271 nm) was taken at time intervals of 5 min until 1 h then time intervals of 10 min for another hour.

All formulations contained 25 mg of naproxen. The reason for choosing 25 mg of naproxen was because of naproxen poor solubility profile at pH 1.2 due to its weak acidic properties. Naproxen would need to be able to dissolve completely at pH 1.2 in order for the dissolution test to be reliable. According to studies by Mora and Martinez (39), naproxen solubility at 35°C and pH 1.2 was 1.16 × 10^−4^ mol/L or 27 mg/L, hence 25 mg used in test seemed reasonable. As for pH 7.4, naproxen was extremely soluble with a solubility of 1.455 × 10^−2^ mol/L or ~3347 mg/L. It should be noted that pH 1.2 sink condition was not maintained and this pH was only used for comparison of various formulations.

### Differential Scanning Calorimetry Studies

Differential scanning calorimetry (DSC) (DCS 4000, Perkin Elmer, USA) was performed on the excipients, pure naproxen, and the chosen formulations with the fastest dissolution rate in order to assess their thermal behavior. Samples weighing between 3 and 6 mg were sealed in aluminum pan and thermal behavior was investigated at a scanning rate of 10°C/min, from 25to 200°C under nitrogen atmosphere.

### X-ray Powder Diffraction Studies

X-ray powder diffraction (XRPD) was performed using an X-ray diffractometer (D5000, Siemens, Germany) on naproxen, excipients, and selected formulations to characterize the solid state of the materials used. Samples were scanned over a range of 2θ at a voltage of 40 kV and current of 30 mA, with a scanning angle ranged from 5^o^ to 40^o^ and a scan rate of 0.2^o^/s.

There were 2 methods of analyzing the % relative crystallinity, which were integrated peak method (Eq. 4) and peak height method (Eq. 5) (40). For integrated peak method, the area under the peak was measured *via* the trapezoid method. In Eq. 4, A_s_ is the integrated peak value of a sample and A_r_ is the integrated peak value of a reference, which is usually the pure API. In Eq. 5, H_s_ is the peak height value of a sample and H_r_ is peak height value of a reference, which is usually the pure API.

4 $$ \%\mathrm{XRD}\ \mathrm{relative}\ \mathrm{crystallinity}=\left({\mathrm{A}}_{\mathrm{s}}/{\mathrm{A}}_{\mathrm{r}}\right)\ \mathrm{x}\ 100 $$

5 $$ \%\mathrm{XRD}\ \mathrm{relative}\ \mathrm{crystallinity}=\left({\mathrm{H}}_{\mathrm{s}}/{\mathrm{H}}_{\mathrm{r}}\right)\ \mathrm{x}\ 100 $$

### Statistical and Mathematical Analysis

Mean cumulative % drug release after 2 h from the dissolution test were statistically analyzed by one-way analysis of variance (ANOVA). Results were quoted as significant where *p* < 0.05.

Specific mathematical equations were used to analyze and compare dissolution profiles, which includes difference factor (f_1,_ Eq. 6) and similarity factor (f_2,_ Eq. 7) as described by Moore and Flanner (41). Both methods have been recommended by the US FDA (Food and drug administration) (42) and implemented by the FDA in various guidance documents (43,44). In brief, f_1_ value between 0 and 15 and f_2_ value between 50 and 100 indicates equivalence of the two dissolution profiles (45). Details of the equations can be found in various literature (42,46–48). The *n* represents the number of dissolution sample times and *R*_*t*_ and *T*_*t*_ represent the mean % of drug dissolved at each time point (*t*).

6 $$ {f}_1=\left\{\left[{\sum}_{t=1}\mathrm{n}|{R}_t-{T}_t|\right]/\left[{\sum}_{t=1}\mathrm{n}\;{R}_t\right]\right\}\bullet 100 $$

7 $$ {f}_2=50\bullet \log \left\{{\left[1+\left(1/\mathrm{n}\right){\sum}_{t=1}\mathrm{n}\;{\left({R}_t-{T}_t\right)}^2\right]}^{-0.5}\bullet 100\right\} $$

## RESULTS AND DISCUSSION

### Solubility Studies

As shown in Table II, naproxen is most soluble in Kolliphor EL liquid vehicle and least soluble in Tween 80. Despite this, the formulation containing Tween 80 (LP-3) unexpectedly showed the fastest dissolution rate at pH 1.2 (this will be discussed later in the manuscript). It is generally thought that formulation containing the liquid vehicle with the highest solubility to the drug would exhibit the fastest drug release rate. This is due to less drugs in crystalline form and more drugs are in solubilized or in molecularly dispersed state in the carrier, thus increasing surface area for dissolution (9).

**Table II:** Solubility of Naproxen in Various Liquid Vehicles at 37°C (*n* = 3)

Non-volatile solvent	Mean concentration (mg/ml) ± SD^a^	Inference
PEG 200	7.88 ± 4.87	Slightly soluble
PG	5.13 ± 0.78	Slightly soluble
Tween 80	2.99 ± 1.01	Slightly soluble
Labrafil	10.73 ± 1.15	Sparingly soluble
Labrasol	5.14 ± 2.44	Slightly soluble
Kolliphor EL	15.83 ± 0.77	Sparingly soluble

For the composition of each formulation, refer to Table I

^a^SD, standard deviation

It is noteworthy to point out that apart from drug solubility, other physicochemical characteristics of liquid vehicles such as lipophilicity, viscosity, polarity, chemical structure, and molecular mass may affect drug release behavior (1). Hence, this may be the reason why the solubility result does not strictly match the drug release result. Nonetheless, the solubility of the drug in a liquid vehicle is a major factor that could greatly influence the drug release profile.

### Extrusion and Spheronization

**Fig. 2:**
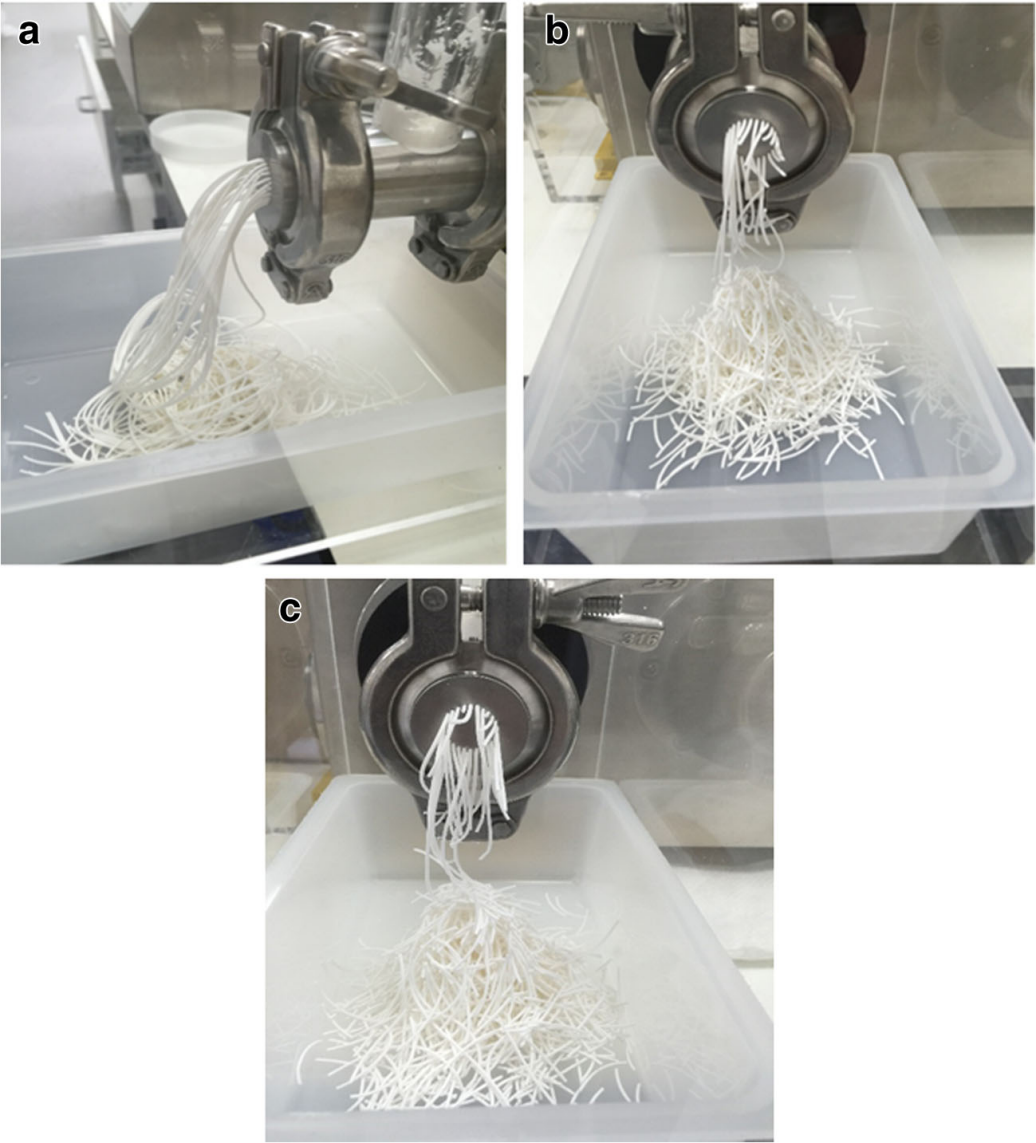
Image (**a**) of extrudate of a formulation (naproxen, Tween 80, Avicel, and Aerosil) containing high water content, exhibiting high plasticity. Image (**b**) of extrudate of a formulation (naproxen, PG, Avicel, and Aerosil) containing lower water content, exhibiting lower plasticity. Image (**c**) of extrudate of physical mixture formulation (naproxen, Avicel, and Aerosil)

**Fig. 3:**
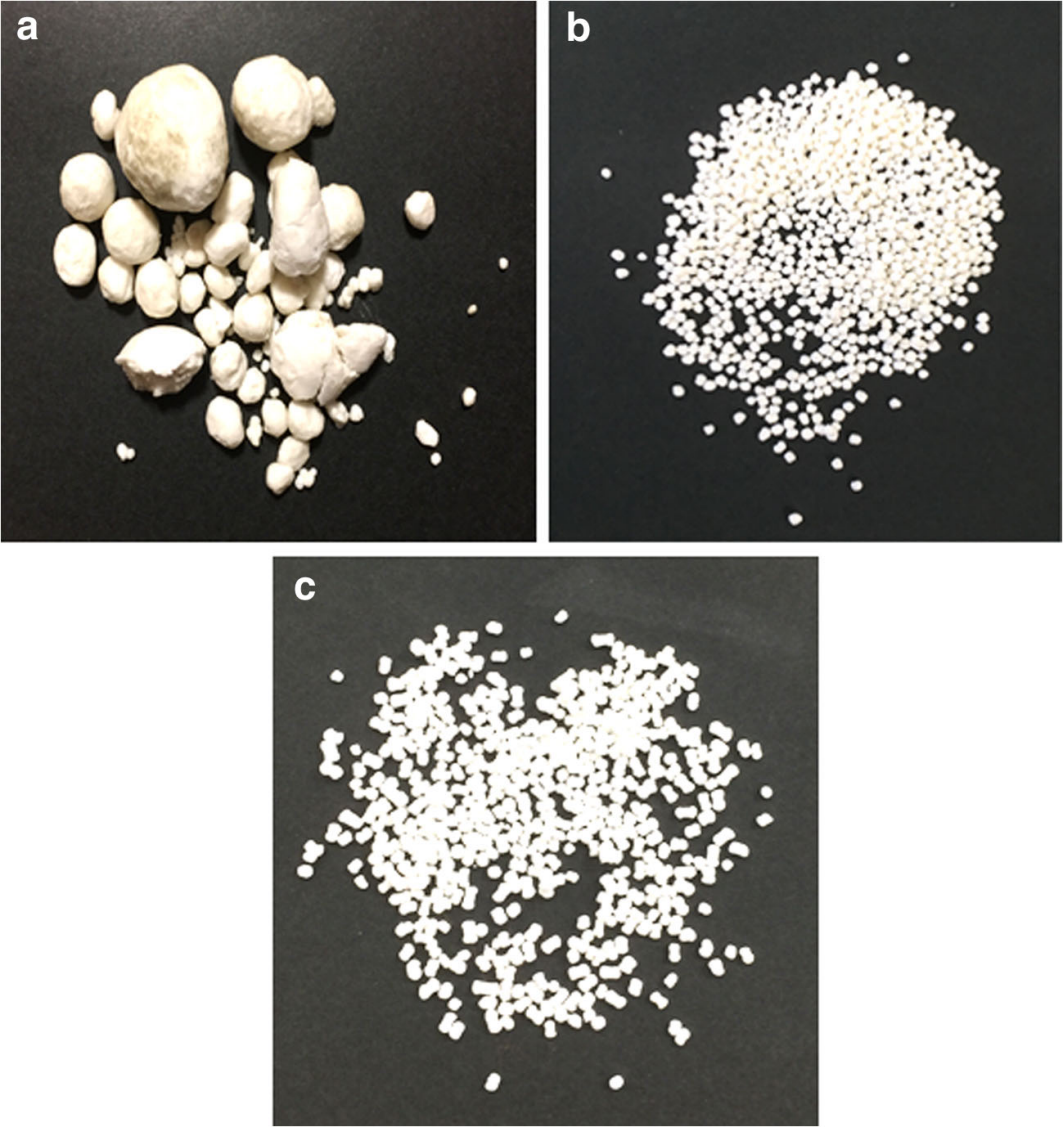
Image (**a**) of agglomerated product after spheronizing a formulation (naproxen, Tween 80, Avicel, and Aerosil) containing high water content and longer threads. Image (**b**) of good quality pellets (spherical particles with less size variation) after spheronizing a formulation (naproxen, PG, Avicel, and Aerosil) containing lower water content and shorter threads. Image (**c**) of reasonable quality pellets (dumbbell shape would be considered less quality but reasonable) of physical mixture formulation (naproxen, Avicel, and Aerosil)

It should be noted that in the preliminary work, the moisture level or plastic property of extrudates greatly affects the success of spheronization. Extrudate plastic property is directly linked to the amount of water added, which is the granulating liquid. The more water added the greater degree of plasticity. When the extrudate’s plasticity reaches above a critical point, it would usually be in a form of long threads rather than short threads (usually 3–5 cm) as shown in Fig. 2a. This extrudate’s degree of plasticity was above the critical point, resulting in agglomeration during the spheronization as shown in Fig. 3a. However, below the critical point, shorter extrudates are formed that successfully spheronized into pellets as shown in Fig. 2b, c and Fig. 3b, c. Thus, finding the optimal water content in extrudate has been seen prudent in making liqui-pellets. In addition, spheronization speed and time should be taken into account as high speed and long duration of spheronization could lead to agglomeration.

It can also be seen in Fig. 3 that the quality of the pellets from formulation (b) can be similar or better than that of pellets without liquid vehicle (c). This could be due to liquid vehicle improving the rheological property of the extrudate to form good spherical pellets.

### Evaluation of Naproxen Liqui-Pellet

#### Liqui-Pellet Flow Property

The results obtained from the flowability studies (Table III) indicate that liqui-pellet is indeed a very promising approach to overcome poor flowability with high liquid load factor, which is one of the biggest hurdles in current liquisolid technology. According to the angle of repose results (Table III), all formulations achieved excellent flow property apart from LP-3, which is in the borderline between excellent to good flow property. CI% results (Table III) show that all formulations achieved excellent flow property. Such results have never been achieved in liquisolid formulation with high liquid load factor before. Although there was an improvement in the flow of liqusolid powders by applying the granulation technique (49), the liquid load factor was much lower than the liquid-pellet in the current study.

**Table III:** Flow Rate (g/s), Angle of Repose and Carr’s Compressible Index (CI%) of All Liqui-Pellet Formulation (*n* = 3)

Formulation^a^	Flow rate (g/s) ± SD^b^	Angle of repose ± SD^b^	CI% ± SD^b^	Inference according to angle of repose	Inference according to CI%
Physical mixture pellet	8.02 ± 0.24	27.95 ± 0.14	9.08 ± 0.87	Excellent flow	Excellent flow
LP-1	8.85 ± 0.16	25.89 ± 0.95	6.07 ± 1.71	Excellent flow	Excellent flow
LP-2	8.88 ± 0.07	23.53 ± 0.19	8.93 ± 0.93	Excellent flow	Excellent flow
LP-3	5.67 ± 0.28	30.26 ± 0.09	3.38 ± 0.71	Excellent-good flow property	Excellent flow property
LP-4	6.64 ± 0.23	27.37 ± 0.21	4.16 ± 1.67	Excellent flow	Excellent flow
LP-5	7.10 ± 0.16	27.52 ± 0.24	3.18 ± 1.58	Excellent flow	Excellent flow
LP-6	7.12 ± 0.07	29.24 ± 0.57	3.42 ± 0.00	Excellent flow	Excellent flow

^a^For the composition of each formulation, refer to Table I

^b^SD, standard deviation from the mean

It is interesting to see that the liquid load factor (L_f_) in liqui-pellet formulations is as high as 1 (Table I), which is considered very high in liquisolid formulations. In fact, 28% of the total mass of the pellets is co-solvent and yet the flow property is excellent. To put this into perspective, a comparison with various studies will be discussed. For example, in studies by Tiong and Elkordy, the naproxen liquisolid composition highest L_f_ was 0.9 with very poor flow property (Carr’s index of 31.58) (35). Even the formulation with L_f_ of 0.168 only had fair flow property (Carr’s index of 20) (35). In studies by Javadzadeh *et al*., it is claimed that with the use of an additive, such as PEG 3500, the L_f_ can be increased (36). They observed an increase of carbamazepine L_f_ from 0.25 to 0.6, which is considered high (36). It can be seen clearly that liqui-pellet L_f_ is much higher than the formulations in the mentioned studies, and yet there are more rooms for liqui-pellet to be optimized such as incorporating polymeric additive. In studies by Hentzschel *et al*., a commonly used carrier (Avicel of specific grade) and coating material (Aerosil of specific grade) were replaced with Neusilin, which had a much larger specific surface area (SSA) than Avicel to make tocopherol acetate liquisolid tablet (50). This large SSA increased the L_f_ from 0.22 to 1.58 (factor of ~ 7); however, it was still limited by its flow property.

With such high L_f_ in liqui-pellet whilst still achieving excellent flow property, the implication for commercial use is very appealing as currently there is no liquisolid formulation in the market. Liquisolid technology in itself has great merits in the advantages it offers, but its drawbacks of poor flowability and larger mass of excipients in dosage form for high dose drug have made it difficult to establish itself for commercial use. In fact, with high L_f_ and excellent flowability, it would seem possible that liqui-pellet can achieve acceptable weight for high dose drug since poor flow property is the key reason for bulky dosage form. Formulations with poor flowability due to liquid medication require more carrier and coating excipients to improve its flow property, thus increasing overall dosage form weight. The improvement of dosage form weight is currently undergoing studies by the authors. It would be fundamentally reasonable to postulate that liqui-pellet is highly commercially feasible without having the inherent advantages of liquisolid formulation compromised.

It is also worth mentioning that due to flow property not being a major drawback in liqui-pellet, this effectively reduces the reliance on the current liquisolid mathematical model introduced by Spireas (4). Flowable liquid-retention potential and compressible liquid-retention need less attention in liqui-pellet. In other words, high L_f_ can be achieved whilst maintaining excellent flow property, and compressibility is not a major factor for pellets in capsules. Other parameters such as R-value and choices of excipients may not need to be compromised by flow property.

Despite results obtained in this study, liqui-pellet and liqui-mass system are still in its infancy; there are still areas for further optimization in order to realize liqui-pellet full potential, which at present is undergoing studies by the authors.

#### Determination of the Amount of API in Liqui-Pellet Formulation

Assay *via* a spectrophotometer (Table IV) shows that all formulations except LP-2 have a good amount of drug nearing to 100%. What is unusual is that LP-2 shows ~ 30% more naproxen than expected. Initially, it is thought that this is due to experimental or processing error and so the LP-2 is remade but the assay still shows ~ 130% drug content. The absorbance of 25 mg naproxen in 900 mL of dissolution medium was increased from 0.47 ± 0.01 to 0.63 ± 0.02 when this dissolution medium contained an additional 40 mg PG (this was exactly how much PG associated with liqui-pellet formulation). The increase in the absorbance value in the presence of PG was calculated to be around 34%. This is in agreement with the dissolution data for LP-2 where the maximum dissolution percentage for LP-2 was around 130% which is 30% higher than expected. This indicates that the presence of PG interferes with the wavelength used to measure naproxen and this is the main reason for having more than 100% drug release (130%) for the samples containing PG. There have been other cases when PG interfers with absorbance reading such as in Dastidar and Sa studies (51), where PG can interfere with the absorbance of diazepam causing an increase in the absorbance reading. It should be pointed out that since PG is not the chosen suitable liquid vehicle for naproxen liqui-pellet, there is no major issue concerning the interaction between naproxen and PG.

**Table IV:** Spectrophotometric Assay (Wavelength 271 nm) Showing % Drug Content in 25 mg Naproxen Formulations and Pure Naproxen Powder (*n* = 3)

Formulation^a^	Mean % drug release ± SD^b^
Pure naproxen powder	98.78 ± 0.23
Physical mixture pellet	96.54 ± 2.30
LP-1	95.68 ± 1.22
LP-2	129.68 ± 0.58
LP-3	100.88 ± 1.10
LP-4	101.84 ± 0.66
LP-5	99.94 ± 0.46
LP-6	101.79 ± 1.27

^a^For the composition of each formulation, refer to Table I

^b^SD, standard deviation from the mean

#### Friability Test

The results obtained from the friability test (Table V) show all formulations having % weight loss below 1%, which is considered acceptable for tablets under USP standard. This indicates that liqui-pellets are ideal for commercial manufacturing as it is robust to friability. The microcrystalline cellulose carrier forms strong bonding within its structure when water is added, producing pellets with a strong structure which is resistant to being friable. Also, the liquid vehicle in liqui-pellet increases the pellet plasticity, which effectively increases the pellet resistant to friability.

**Table V:** Weight Loss of 3 g of Each Formulation Under Rotational Speed of 25 rpm for 4 min

Formulation	% weight loss
Physical mixture pellet	0.54
LP-1	0.03
LP-2	0.00
LP-3	0.29
LP-4	0.53
LP-5	0.30
LP-6	0.43

#### Particle Size of Liqui-Pellet *via* Sieve Method

**Fig. 4:**
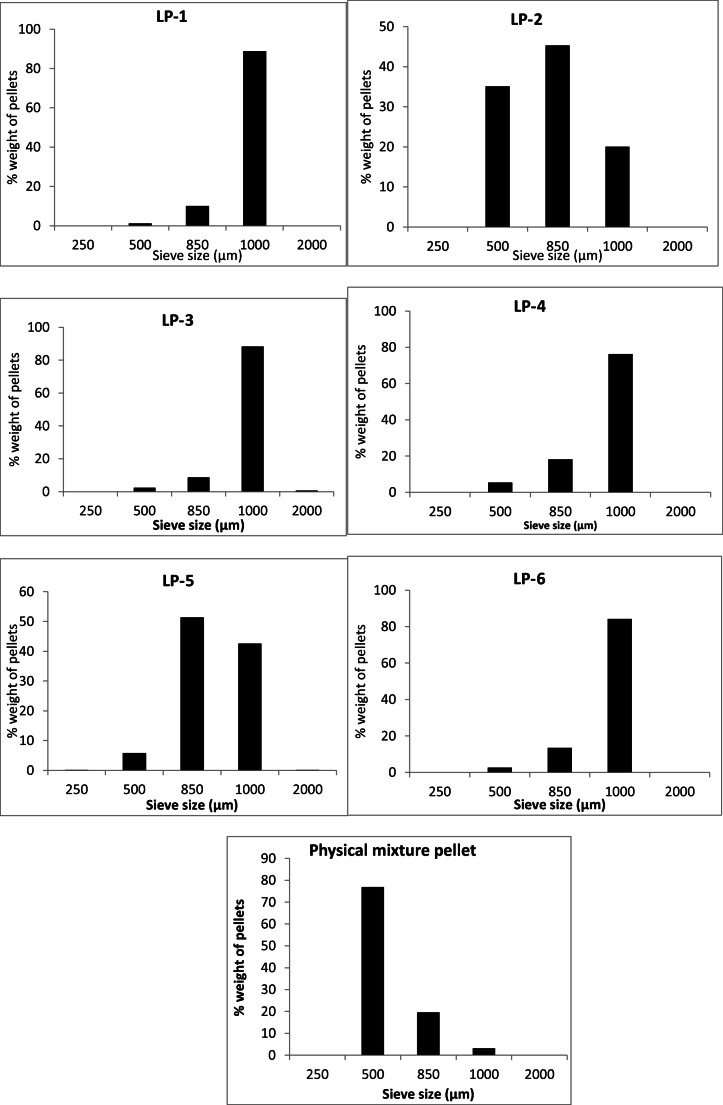
Graph showing particle size distribution (PSD) of all formulations *via* sieve method

In Fig. 4, it is clear that all formulations are mostly below 2 mm in size. Formulations LP-1, LP-3, LP-4, and LP-6 are mostly within 1 mm. This shows that it is possible to produce uniform size of liqui-pellet, which is important in regard to quality control in manufacturing for commercial use.

Formulations LP-2 and LP-5 have a broader size distribution with smaller size pellets compared to the rest of the liqui-pellet formulations. In regard to formulation LP-2, ~ 45% and ~ 35% of total pellet fall within 850 μm and 500 μm respectively. As for formulation LP-5, ~ 51% and ~ 5.8% of total pellet fall within 850 μm and 500 μm respectively. This indicates the liquid vehicle can have an effect on liqui-pellet size distribution, which can be assumed to be due to its effect on extrudate plasticity. As for physical mixture pellet, which does not contain liquid vehicle, ~ 77% of total pellet are within 500 μm. Hence, it seems to indicate liquid vehicle tends to increase pellet size.

Since all of the pellets are almost entirely equal or less than 2 mm, it will be emptied from the stomach into the small intestine relatively fast, similar to how liquid is emptied (52). This is advantageous for weakly acidic drugs (*i.e.*, naproxen), as they undergo dissolution at a faster rate in less acidic and more alkaline environment such as the small intestine. Hence, it is suggested that the bioavailability and speed of drug absorption may improve.

#### Stereoscopic Analysis

The Feret’s diameter, roundness, and elongation ratio were calculated and shown in Table VI. In general, the Feret’s diameter of the pellet seems to agree with most of the results from particle size analysis. Thus, supporting the claim that different liquid vehicles can influence pellet size and generally increases the pellet size. However, there are some discrepancies between the stereoscopic and particle size analysis. It can be seen that the mean Ferret diameter of the physical mixture pellet, LP-2, and LP-5 could be overestimated. In fact, since the pellets are not perfectly spherical and are usually in the most stable orientation, meaning that the smallest dimension is orientated vertically; therefore, overestimation is likely to occur (53). In reality, it is actually difficult to attain perfectly spherical particles.

**Table VI:** Stereoscopic Analysis Showing the Mean Feret’s Diameter, Mean Roundness, and Mean Elongation of Each Formulation (*n* = 100)

Formulations^a^	Mean Feret’s diameter (mm)	Mean roundness ± SD^b^	Mean elongation ratio ± SD^b^
Physical mixture pellet	1.028	1.25 ± 0.12	1.41 ± 0.19
LP-1	1.294	1.12 ± 0.07	1.15 ± 0.10
LP-2	1.000	1.12 ± 0.03	1.17 ± 0.09
LP-3	1.517	1.14 ± 0.09	1.18 ± 0.17
LP-4	1.303	1.18 ± 0.07	1.25 ± 0.11
LP-5	1.268	1.24 ± 0.11	1.41 ± 0.21
LP-6	1.535	1.38 ± 0.18	1.47 ± 0.18

^a^For the composition of each formulation, refer to Table I

^b^SD, standard deviation from the mean

Formulations LP-6, LP-5, and physical mixture pellet showed the least roundness and largest elongation ratio. Among them, LP-6 has the highest deviation from perfect roundness (1.38) and largest mean elongation ratio (1.47). Nevertheless, the pellets are good enough to achieve excellent flowability (Table III). As for the rest of the formulations, the results seem to suggest the rest of the liqui-pellets have good roundness and minimal elongation.

#### Morphological Studies on Pellets *via* SEM

**Fig. 5:**
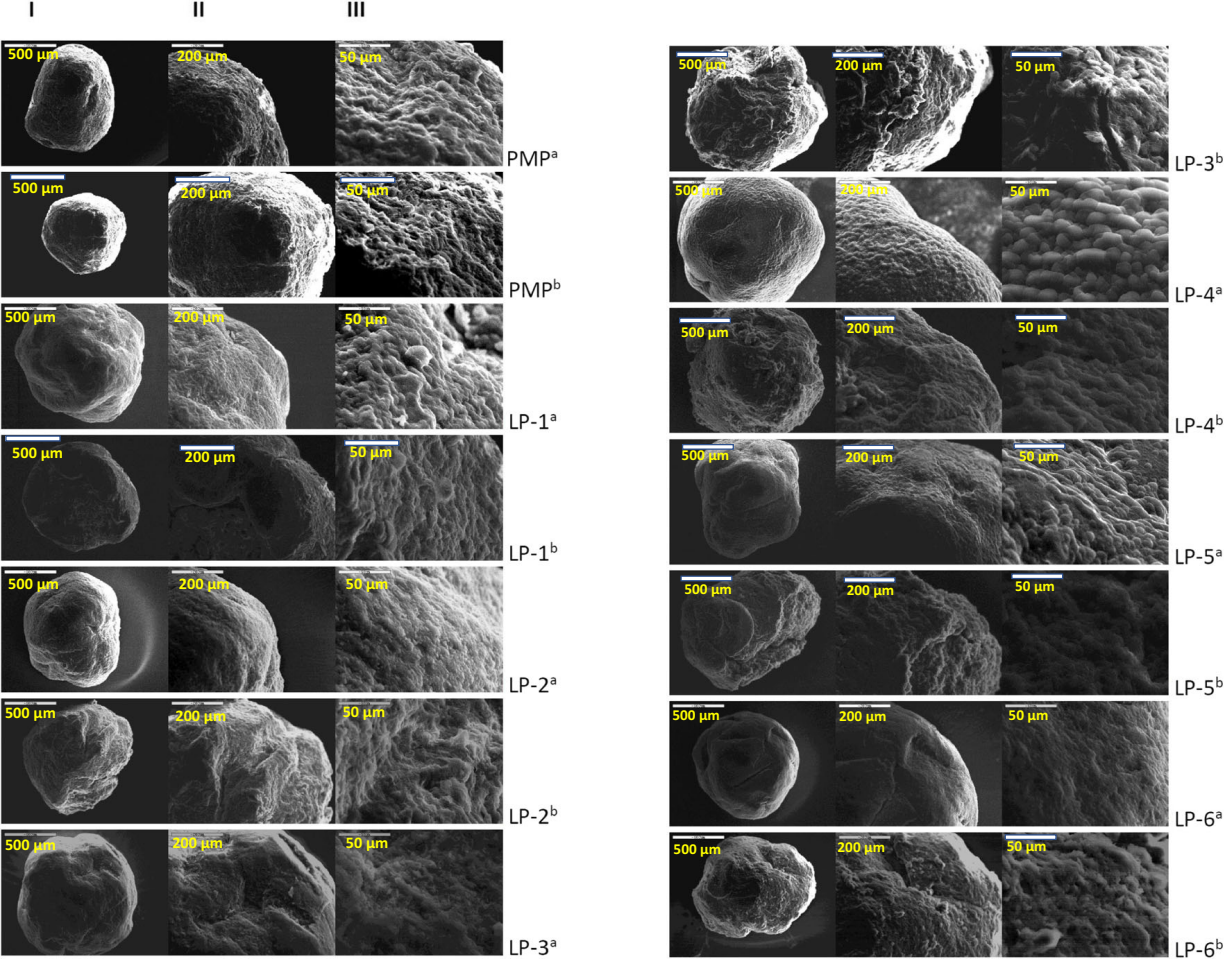
Images from SEM of all formulation; **I**. × 80 magnification, **II**. × 200 magnification, and **III**. × 800 magnification. Note ^a^ refers to pellet before undergoing dissolution test and ^b^ SD refers to after dissolution test

According to Fig. 5, it can be seen that physical mixture pellet (PMP) has a rougher surface structure than most of the formulations. This seems to suggest that the co-solvent in liqui-pellet formulations has an influence on the pellet surface morphology, which generally results in a smoother surface. It can be speculated that the dissolved crystals of naproxen in its respective liquid vehicle have contributed to reducing the overall crystallinity of the pellet. Given that all formulations resisted disintegration after the dissolution test, surface morphology after dissolution test was observed. The physical mixture pellet surface structure is not much different from before the dissolution test. As for most of the formulation containing different types of co-solvent, it is observed that their surface structure became rougher after undergoing dissolution test. Since around 49% of the formulated liqui-pellet is liquid medication, it is reasonable to suspect the increase in roughness could be due to liquid medication moving out from the pellet into the dissolution medium, resulting the pellet reverting back to a more crystalline structure or shrinking into a rougher surface structure. Also, there may have been slight disintegration around the surface when the liquid medication leaves the pellet. The fact that it is possible to study the morphology of the pellets after undergoing dissolution test demonstrates the strong bonding within the microcrystalline cellulose structure, rendering the pellet non-disintegrating.

When observing liqui-pellet surface structure before the dissolution test (Fig. 5), it can be observed that different liquid vehicles give a different surface structure. Formulations containing PEG 200 (LP-1) or Tween 80 (LP-3) have similar surface structure and both produce relatively rough surface in comparison to the other liqui-pellet formulations (Fig. 5). Such surface structure is different from the formulation containing PG (LP-2) or Kolliphor EL (LP-6) where both produce smooth surface liqui-pellet. Liqui-pellet containing Labrafil (LP-4) and Labrasol (LP-5) has a similar surface structure to one another. As shown in Fig. 5, both of their surface structure are relatively smooth but interestingly have a smooth round bump that resembles micron-size pebbles. It will be interesting to see how these various surface morphologies may have an impact on the success of applying coating techniques in these pellets in future studies.

The results from the morphology studies and dissolution studies show no significant correlation between surface structures affecting the dissolution rate. This could be due to the drug release rate being affected by many additional factors as well as surface properties including drug solubility and physicochemical characteristics of liquid vehicle (1). Hence, this may be the reason why it does not appear to be clear how surface structure affects the drug release rate. Further studies on the surface structure are needed on liqui-pellet to determine its impact on drug release.

#### Drug Release Study

**Fig. 6:**
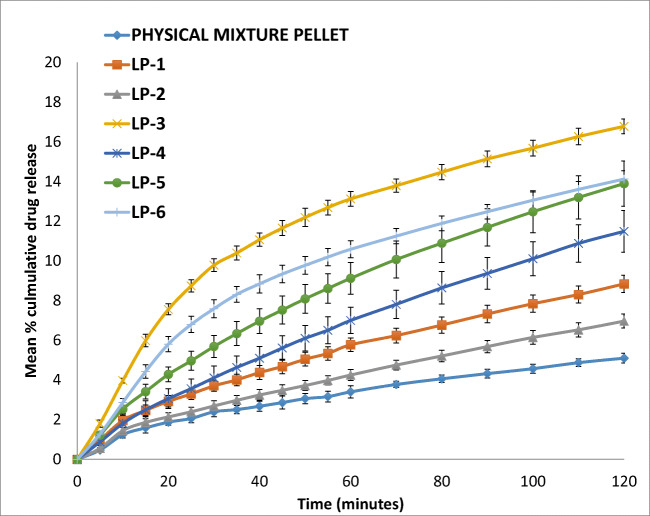
Dissolution profile of pellets in capsule for naproxen 25 mg with various liquid vehicles and physical mixture pellet (pH 1.2)

**Fig. 7:**
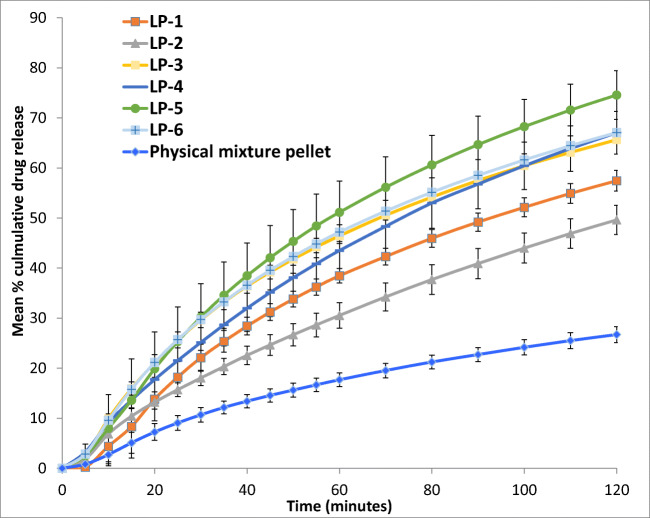
Dissolution profile of pellets in capsule for naproxen 25 mg with various liquid vehicles and physical mixture pellet (pH 7.4)

The dissolution profiles of all formulations at pH 1.2 are shown in Fig. 6. It should be noted that, although naproxen is poorly soluble in acidic condition and the dissolution test should be carried out at higher pH or with sink condition, for comparison purpose, the dissolution of liqui-pellets was initially carried out at pH 1.2. It can be seen clearly in Figs. 6 and 7 that liquid vehicle causes considerable enhancement of drug release rate compared to physical mixture pellet (*p* < 0.05), which does not contain a liquid vehicle. The difference factor (F_1_) and similarity factor (F_2_) of the best formulation (LP-3) and physical mixture at acidic condition are 73.16 and 53.53 respectively. As seen, the F_1_ value indicates a marked difference in dissolution profile. The *p* value and F_1_ value indicate that there is a difference in dissolution profile. The enhanced drug release *via* API solubilized or held at molecularly dispersed state is maintained even after extrusion and spheronization. This demonstrates that the enhanced drug release mechanism *via* liquisolid concept can be combined with the pelletization technique mentioned earlier.

Despite the solubility test (Table II), the dissolution results at pH 1.2 (Fig. 6) show formulation with Tween 80 (LP-3) achieving the fastest drug release rate in comparison to the other formulations with different liquid vehicles. Tween 80 has the fastest drug release rate followed by Kolliphor EL (LP-6) then Labrasol (LP-5). Admittedly, even though the formulation with Tween 80 has the fastest dissolution rate, the percentage cumulative drug release is about 17% after 2 h, which is poor. Nonetheless, the poor dissolution rate is expected as naproxen is poorly soluble under acidic pH (37) and the microcrystalline-based pellets are not suitable for fast release formulation due to resistance to disintegration (54).

It is obvious that drug release rate increases significantly at pH 7.4 (Fig. 7); however, what is interesting is that the formulation containing Labrasol (LP-5) has the fastest drug release rate of ~ 75% after 2 h instead of Tween 80 (LP-3), which is ~ 66% after 2 h. This shows a different trend compared to the results when the pH is 1.2. Their F_1_ and F_2_ are 9.23 and 66.04 respectively, indicating little difference in dissolution profile. Also, at pH 7.4, Tween 80 (LP-3) and Kolliphor EL (LP-6) dissolution profile are almost identical (F_1_ = 1.43 and F_2_ = 95.66). Furthermore, around 90 min during the dissolution test, Labrafil (LP-4) drug release profile is similar to that of Tween 80 and Kolliphor EL. Such changes in dissolution profile in comparison to the results obtained at pH 1.2 suggest that pH can affect different liquid vehicles’ influence on drug release rate, and the degree of this effect depends on the choice of liquid vehicle. Thus, the effect of pH on different liquid vehicles seems fundamental for future studies, particularly in choosing the most appropriate co-solvent for a specific region of the gastrointestinal tract which the drug was supposed to be released most efficiently in. In this case, the authors believe that Tween 80 is the most suitable liquid vehicle since the aim is to have a fast onset of action and fast drug release rate. Thus, it is prudent that the drug release rate is high in acidic condition as the drug will be in the stomach before entering the small intestine. It should be noted that although the aim is to have a fast onset of action and fast drug release, in reality, a drug like naproxen would have enteric coat due to potential GI irritation and the main site of absorption will be in the small intestine.

Despite Labrasol having the best drug release at pH 7.4, Tween 80 drug release rate is only ~ 10% lower than Labrasol. Nonetheless, Labrasol and Kolliphor EL may also be a suitable liquid vehicle for naproxen. In studies by Tiong and Elkordy, liquisolid tablet containing Kolliphor EL (formerly known as cremophor EL) and naproxen of 20% *w*/*w* gave the fastest release, confirming that Kolliphor EL may also be a suitable liquid vehicle for naproxen (35).

#### DSC Studies

**Fig. 8:**
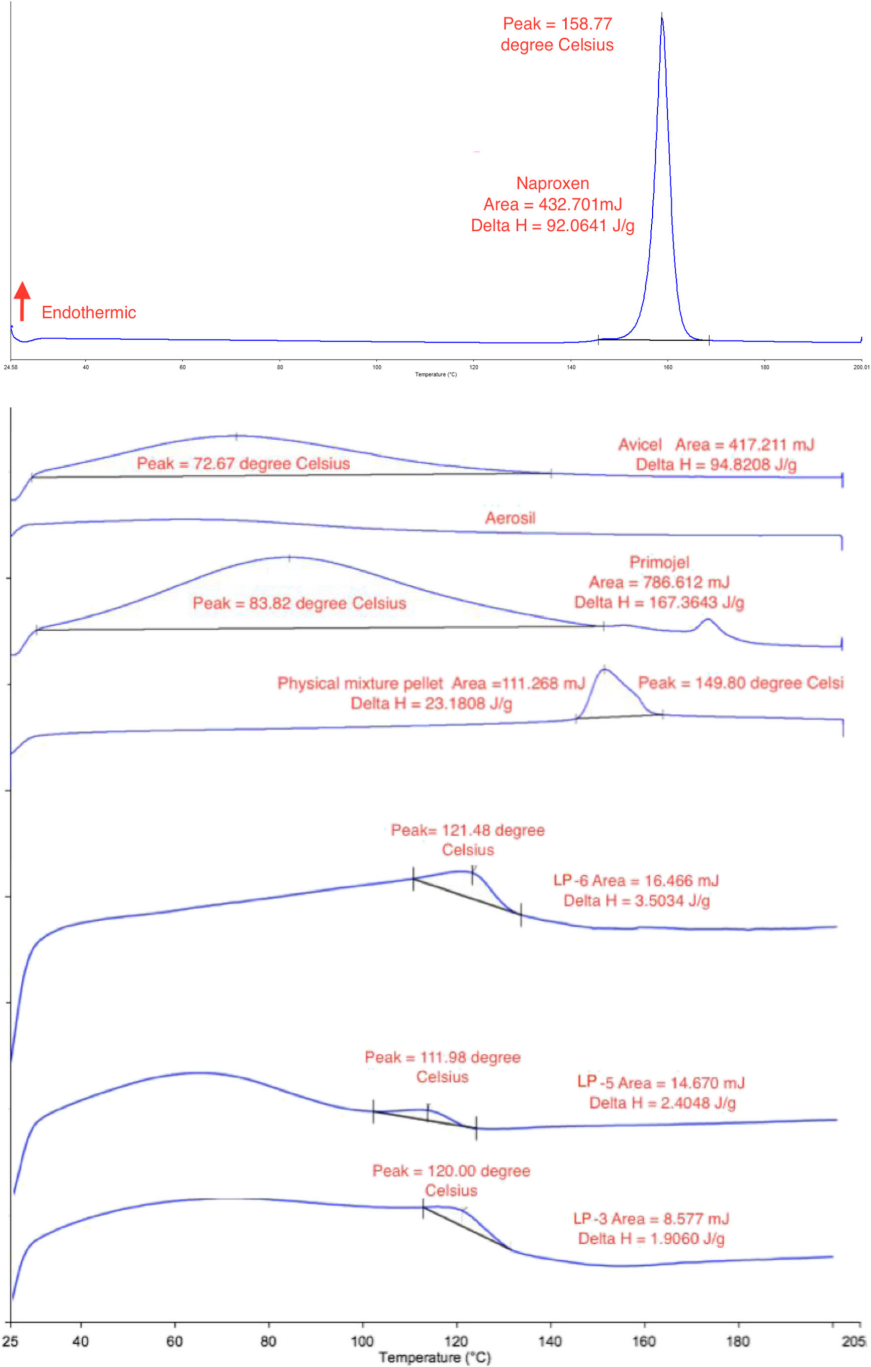
DSC traces of Avicel, Aerosil, physical mixture pellet, LP-3, LP-5, and LP-6. Note, the scales of excipients and formulations are different in order to show the peak more clearly

Thermogram obtained from the DSC includes naproxen, Avicel, Aerosil, Primojel, physical mixture pellets, LP-3, LP-5, and LP-6 (Fig. 8). There is a sharp endothermic peak (T_m_ = 158.77°C and ΔH = 92.06 J/g) for naproxen, indicating its crystalline state. Avicel (T_m_ = 72.67°C and ΔH = 94.82 J/g) and Primojel (T_m_ = 83.82°C and ΔH = 167.36 J/g) show broad peaks which could be due to water within Avicel and Primojel evaporating, as they are hygroscopic materials. The evaporation of water is also observed by Tiong and Elkordy (48). Aerosil had no definitive peak.

**Fig. 9:**
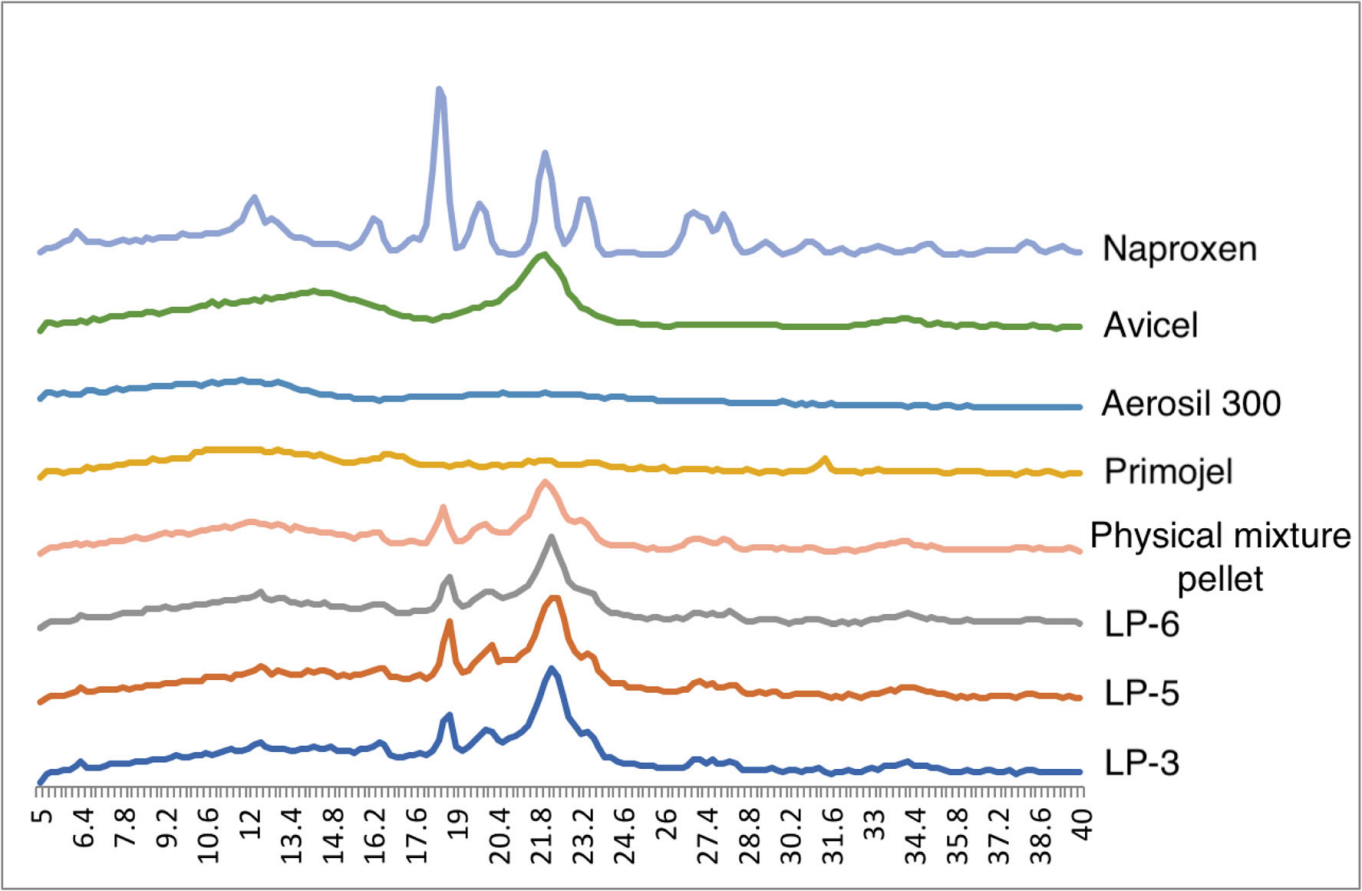
Diffractograms of naproxen, Avicel, Aerosil, physical mixture pellet, LP-3, LP-5, and LP-6

In the physical mixture pellet trace (Fig. 8), the peak is at a lower temperature than naproxen (Fig. 8). The peak shifts from 158.77°C (Fig. 8) to 149.80°C (Fig. 9), which could be due to the Avicel affecting the overall peak of naproxen in the physical mixture pellets. Nevertheless, the crystalline state of naproxen is still present.

However, in formulation LP-3 (T_m_ = 120°C and ΔH = 1.9060 J/g), LP-5 (T_m_ = 111.98°C and ΔH = 2.4048 J/g), and LP-6 (T_m_ = 121.48°C and ΔH = 3.5034 J/g), the DSC traces show the absence of naproxen peak and that T_m_ is lower than physical mixture. This indicates that liqui-pellets have reduced crystallinity and possibly have become more amorphous; thus, the improvement in dissolution.

#### XRPD Studies

Naproxen has major peaks at 2θ values of 12.2, 16.2, 18.4, 19.6, 22.2, 23.2, 26.8, and 27.8^o^ (Fig. 9). These peaks are similar to the naproxen diffractogram in Maghsoodi studies (55). However, the differences are in the presence of a sharp peak at ~ 7^o^ and the absence of peak at 26.8^o^ compared to Maghsoodi studies. Naproxen peaks are also similar to naproxen diffractogram in Mello and Ricci-Junior studies (56), but again, there are some differences too. This could be due to a different scan rate settings. Nonetheless, the main diagnostic peaks of naproxen are present.

The physical mixture and the chosen formulation (LP-3, LP-5, and LP-6) diffractograms (Fig. 9) have no peaks other than that of naproxen and Avicel, which indicates no interaction between the excipients and the API. It is clear from Fig. 9 that physical mixture and formulation LP-3, LP-5, and LP-6 have reduced crystallinity compared to the pure naproxen, agreeing with the result observed in the DSC test. The reduced crystallinity partially could be due to the presence of Avicel in the samples particularly in the case of physical mixtures as the drug should be in crystalline state. In the case of liquid-pellet formulation, it could also be due to molecularly dispersion of naproxen. From Fig. 9, it is difficult to say physical mixture is more crystalline compared to liquid-pellet formulations. This is unusual and unexpected as it was assumed that liqui-pellet would be less crystalline than the physical mixture pellet due to the liquid vehicle dissolving the crystalline API. It is thought that perhaps the amorphous nature of some of the excipients may have overlapped naproxen sharp peaks in the physical mixtures. Also, other factors such as inconsistency in particle size and amount of sample place for XRPD may have influenced the diffraction peak.

## CONCLUSION

The emerging next-generation oral dosage form, liqui-pellet, has shown remarkable results in terms of overcoming the major drawbacks in liquisolid technology. Liqui-pellet is able to achieve high liquid load factor whilst maintaining excellent flow property, which has never been achieved in liquisolid technology before. Excellent-good flow property was obtained from all liqui-pellet formulations which had liquid load factor of 1. This is one of the key factors for the observed improvement in enhanced drug release. With major drawbacks of liquisolid technology being overcome using liqui-pellet, the liqui-pellet is anticipated as a highly commercially feasible product. Furthermore, there is potential for further optimization as parameters, such as R-value, and choices of excipients may not need to be compromised by flow property.
